# Transmission of Classical Swine Fever Virus in Cohabitating Piglets with Various Immune Statuses Following Attenuated Live Vaccine

**DOI:** 10.3390/ani13030368

**Published:** 2023-01-21

**Authors:** Chia-Yi Chang, Kuo-Jung Tsai, Ming-Chung Deng, Fun-In Wang, Hsin-Meng Liu, Shu-Hui Tsai, Yang-Chang Tu, Nien-Nong Lin, Yu-Liang Huang

**Affiliations:** 1 School of Veterinary Medicine, National Taiwan University, No. 1, Section 4, Roosevelt Road, Taipei 10617, Taiwan; 2 Animal Health Research Institute, Council of Agriculture, Executive Yuan, 376 Chung-Cheng Road, Tansui, New Taipei City 25158, Taiwan; 3 Bureau of Animal and Plant Health Inspection and Quarantine, Council of Agriculture, Executive Yuan, 9F., No. 100, Sec. 2, Heping-West Road, Zhongzheng Dist., Taipei 10060, Taiwan

**Keywords:** classical swine fever, modified live vaccine, maternally derived antibody, transmission

## Abstract

**Simple Summary:**

Classical swine fever is a highly dangerous pathogen affecting domestic pigs. Vaccination with the modified live vaccine is critical for preventing and controlling classical swine fever. However, many factors, such as maternally derived antibodies via colostrum, could interfere with live vaccine efficacy, leading to incomplete protection in commercial herds. In this study, we investigated the transmission of classical swine fever virus in experimental piglets with various post-vaccination immune statuses. A specific-pathogen-free piglet infected with classical swine fever virus served as the viral donor and primary invader, and was cohabited with piglets with maternally derived antibodies that had or had not undergone vaccination. According to the results, most of the piglets with maternally derived antibodies that were vaccinated were fully protected from contact transmission from the viral donor and blocked viral transmission to the third party (those piglets secondarily exposed through cohabitation). Cell-mediated immunity, represented by specific interferon-γ-secreting cells, served as the key to the viral clearance and recovery. Contrarily, the unvaccinated piglets with low levels of maternally derived antibodies had accelerated classical swine fever virus infection following viral invasion. In conclusion, vaccination still induces solid immunity in commercial herds under maternally derived antibody interference and can block viral transmission in herds.

**Abstract:**

Classical swine fever (CSF) is a systemic hemorrhagic disease affecting domestic pigs and wild boars. The modified live vaccine (MLV) induces quick and solid protection against CSF virus (CSFV) infection. Maternally derived antibodies (MDAs) via colostrum could interfere with the MLV’s efficacy, leading to incomplete protection against CSFV infection for pigs. This study investigated CSFV transmission among experimental piglets with various post-MLV immune statuses. Nineteen piglets, 18 with MDAs and 1 specific-pathogen-free piglet infected with CSFV that served as the CSFV donor, were cohabited with piglets that had or had not been administered the MLV. Five-sixths of the piglets with MDAs that had been administered one dose of MLV were fully protected from contact transmission from the CSFV donor and did not transmit CSFV to the piglets secondarily exposed through cohabitation. Cell-mediated immunity, represented by the anti-CSFV-specific interferon-γ-secreting cells, was key to viral clearance and recovery. After cohabitation with a CSFV donor, the unvaccinated piglets with low MDA levels exhibited CSFV infection and spread CSFV to other piglets through contact; those with high MDA levels recovered but acted as asymptomatic carriers. In conclusion, MLV still induces solid immunity in commercial herds under MDA interference and blocks CSFV transmission within these herds.

## 1. Introduction

Classical swine fever (CSF) is a transboundary, highly contagious, hemorrhagic disease that affects domestic pigs and wild boars and is caused by the classical swine fever virus (CSFV) [1,2]. CSF is a notifiable disease by the World Organisation for Animal Health (WOAH), and CSF is still an endemic disease in Asia, South America, Central America, and the Caribbean. Only North American, Oceanian, and Western European countries have successfully eradicated CSF [3,4,5,6].

CSFV is an enveloped single-strain RNA virus of the *Pestivirus* genus of the Flaviviridae family that also comprises the bovine viral diarrhea virus and border disease virus [1,2]. The viral genome contains approximately 12.3 kb and encodes 3898 amino acids of polyprotein, which are later processed into four structural (C, E^rns^, E1, and E2) and eight nonstructural (N^pro^, p7, NS2, NS3, NS4A, NS4B, NS5A, and NS5B) proteins by viral and cellular proteases [7]. Based on the E2 or NS5B sequences, CSFV is classified into three genotypes with three or four subtypes (1.1, 1.2, 1.3; 2.1, 2.2, 2.3; 3.1, 3.2, 3.3, 3.4) [8].

Depending on CSFV virulence and pig factors, including the age, breed, health condition, and immune status, CSFV-infected pigs can exhibit either acute, subacute, or chronic clinical signs [9,10,11]. Pigs infected with high-virulence CSFV present acute fever and severe hemorrhagic lesions and excrete high viral loads in their feces, saliva, and secretions during their short days of survival. By contrast, pigs infected with moderate-virulence and low-virulence CSFV present subacute and chronic clinical signs and milder lesions and excrete moderate–low levels of CSFV in their feces, saliva, and secretions during their relatively longer days of survival [12,13]. In the field, primarily infected pigs can play a prominent role in the secondary transmission of CSFV to other pigs with variable immunity to CSFV. The transmissibility of moderate-virulence and high-virulence CSFV is higher than that of low-virulence CSFV [12,13].

Vaccination is crucial for the prevention and control of CSF in endemic countries. The modified live vaccine (MLV) is a high-efficacy, safe, and affordable vaccine that is the most widely used among commercial herds. The MLV can quickly induce immunity to provide partial protection at 3 days post-vaccination, and complete protection at 5 days post-vaccination [14,15,16]. However, many factors, including vaccine quality and pigs’ health status and level of maternally derived antibodies (MDAs), can reduce the MLV’s efficacy, leading to the incomplete protection of vaccinated pigs [14,15,16]. In CSF-endemic herds, the timing of MLV vaccination depends on pigs’ MDA levels; high levels of MDAs interfere with the MLV’s efficacy, and low MDA levels increase piglets’ risk of infection [17,18]. Therefore, the formulations of MLV vaccination programs in herds must be based on declines in the levels of MDAs.

A regularly implemented vaccination program against CSF in Taiwan involves vaccinating pregnant sows to provide MDAs through their colostrum to the neonates and then administering the first dose of MLV to the piglets at 3–12 weeks of age. According to surveillance in Taiwan, the average titers of anti-CSFV neutralizing antibodies (NAs) of MDAs in piglets at the time of their first MLV vaccination are higher than 1:32 [19], which could impair the MLV’s efficacy and result in various immune statuses in herds. This study describes an animal experiment to examine the ability of MLV to protect piglets with varying levels of MDAs after cohabitation with a CSFV-infected piglet that acted as a CSFV donor (i.e., the ‘primary invader’).

## 2. Materials and Methods

### 2.1. Cells and Virus

Porcine-circovirus-type-1-free porcine kidney-15 (PK-15) cells were cultured in minimum essential medium (Thermo Fisher Scientific, Carlsbad, CA, USA) with 5% fetal bovine serum (FBS) and incubated at 37 °C in 5% CO_2_. The PK-15 cells supported CSFV propagation, including the Lapinized Philippines Coronel (LPC) vaccine strain of the genotype 1.1 and the TD/96 strain of the genotype 2.1. The virus titers of the LPC and TD/96 strains were 10^7.71^ and 10^5.87^ tissue culture infectious dose 50% (TCID_50_), respectively.

### 2.2. Experimental Design

**Figure 1:**
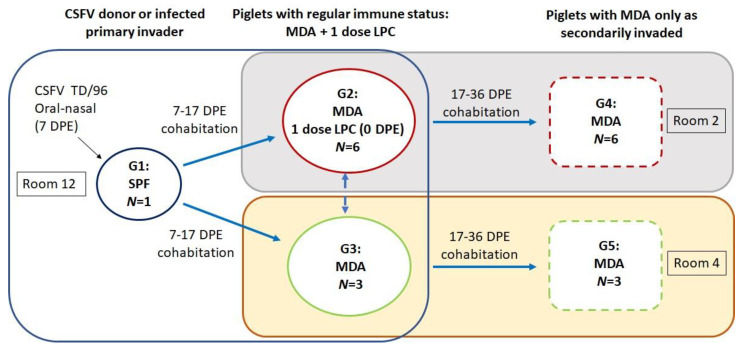
Experimental design.

A total of 19 4-week-old piglets comprising 1 specific-pathogen-free (SPF) piglet without anti-CSFV NAs (Group 1) and 18 healthy piglets (Groups 2–5) from the LPC-vaccinated sows of a CSFV-free herd (Figure 1) were used in the experiment. The 18 healthy piglets were randomly divided into four groups (Groups 2–5); the means of the anti-LPC NA titer (log_2_) among Groups 2–5 were, respectively, 3.7 ± 2.2, 3.7 ± 2.3, 4.3 ± 2.2, and 4.3 ± 1.3 log_2_ folds and were not significantly different at 0 days post-experiment (DPE). The SPF piglet (Group 1) was inoculated oronasally by using the 5 × 10^5^ TCID_50_ TD/96 strain at 7 DPE and served as the primary invader or CSFV donor when cohabiting in Room 12 with Groups 2 and 3 (Figure 1). To test whether the MDAs reduced the efficacy of the MLV, Group 2 (*n* = 6) was vaccinated with a single dose of LPC vaccine (over 1 × 10^4^ TCID_50_/dose) at 0 DPE. To test the MDA protection ability, Group 3 (the control for Group 2; *n* = 3) was not vaccinated with the LPC vaccine, and these piglets thus exhibited MDA decay after 7 days following first contact with the primary invader. Group 1 (primary invader) cohabited with Groups 2 and 3 for 10 days (from 7 to 17 DPE). Group 2 was then transferred to Room 2 to serve as secondary invaders through cohabitation with Group 4 (*n* = 6) from 17 to 36 DPE. Group 3 was transferred to Room 4 to cohabit with Group 5 (*n* = 3). Groups 4 and 5 were thus piglets with 17 days of MDA decay following first contact with the secondary invaders. The Institutional Animal Care and Use Committee of the Animal Health Research Institute approved this animal experiment (approval number A09007).

The animals were monitored daily for clinical signs, and each parameter was scored from 0 to 3, representing normal to severe, according to Mittelholzer’s method [19]. Rectal temperature was measured, and blood, saliva, and fecal samples were collected twice weekly until 35 DPE. Samples were analyzed for CSFV loads and anti-CSFV antibodies. A necropsy was performed at 18 DPE for Group 1, at 36 DPE for Groups 2 and 3, and at 39 DPE for Groups 4 and 5. The environmental samples (i.e., swabs from the fence, feces on the floor, the feed trough, and the drinking fountain) were also analyzed for CSFV.

### 2.3. Quantitative Reverse Transcription Real-Time Polymerase Chain Reaction (QRRT-PCR) of CSFV 

The viral RNAs of the samples were extracted by the QIAamp^®^ Viral RNA Mini Kit (QIAGEN, Hilden, Germany), and were detected through quantitative reverse transcription polymerase chain reaction (QRRT-PCR) [20]. The QRRT-PCR was used to detect the various genotypes and quantitatively assay the CSFV loads of the samples.

### 2.4. Anti-CSFV NAs

Heating of the piglets’ sera to 56 °C inactivated complement and enabled the detection of anti-LPC and TD/96 NAs. In brief, 2-fold serially diluted serum samples, beginning at 1:4, were mixed with equal volumes of 100 TCID_50_ of the LPC or TD/96 strain. The mixtures were incubated at 37 °C for 1 h and subsequently transferred to PK-15 cells in 96-well plates. After incubation for 3 days, the cells were fixed and stained to detect the presence of CSFV antigen through indirect fluorescence assay. The neutralizing titer of the antibody is the log_2_ of the dilution factor of the antibody (reciprocal of dilution) when 50% of the wells are protected from infection.

### 2.5. CSFV-Specific Interferon (IFN)-γ-Secreting Cells

To evaluate the anti-CSFV cell-mediated immunity (CMI) of the piglets, an ex vivo CSFV-specific IFN-γ response test of the peripheral blood mononuclear cells (PBMCs) was performed. The PBMCs from the EDTA-anticoagulated blood were subjected to centrifugation at 400× *g* by using Histopaque-1077 (Sigma-Aldrich, St. Louis, MO, USA). The PBMCs were suspended in RPMI 1640 medium (Thermo Fisher Scientific, Carlsbad, CA, USA) containing 10% (*vol*/*vol*) heat-inactivated FBS, 100 units/mL penicillin G, 100 μg/mL streptomycin, and 0.25 μg/mL amphotericin B. The number of CSFV-specific IFN-γ-secreting PBMCs was detected through porcine IFN-γ single-color enzymatic ELISPOT assay (CTL, Shaker Heights, OH, USA). In 96-well plates, PBMCs at 5 × 10^6^ cells/mL at 100 μL per well with porcine IFN-γ capture antibody were allocated to the mock group (inoculated with RPMI 1640 medium), the TD/96 group (infected with 0.1 MOI), and the ConA group (supplemented with 5 μg/mL concanavalin A; Sigma-Aldrich, St. Louis, MO, USA), serving as the positive control. Duplex stimulations were employed each time. After 48 h of stimulation, the wells were washed, the IFN-γ-detected antibody was bound, and the substrate was seeded. The spots in each well were screened and assayed using the CTL Analyzer.

### 2.6. Immunohistochemistry

The lymphoid tissues were examined through immunohistochemical assay by using the Super Sensitive Polymer HRP IHC detection system (BioGenex, The Hague, The Netherlands) and the 1C7A1 monoclonal antibody against the genotype 2.1 CSFV strains [21]. The development of a brown color indicates the presence of CSFV.

### 2.7. Statistics

An analysis of variance and Duncan’s multiple range test were used to determine the statistically significant differences among groups. Data analysis was performed using SAS (SAS Institute, Cary, NC, USA). A *p* value of less than 0.05 indicated statistical significance.

## 3. Results

### 3.1. Clinical Signs

All piglets were healthy before the experiment (Table 1). The Group 1 piglet was the CSFV donor (primary invader) and was inoculated with the TD/96 strain. The fever and CSFV-associated clinical signs in Group 1 were detected at 12 DPE and 13 DPE, respectively, with continually increasing scores from 13–18 DPE that peaked with a score of 20 at 18 DPE. For the piglets in Groups 2 and 3 that cohabited with the CSFV donor, Group 2, which consisted of the piglets with MDAs that had been vaccinated with the LPC vaccine, were healthy and exhibited no fever or any CSFV-associated clinical signs during the experiment. However, those in Group 3, which comprised the piglets with MDAs that had not undergone LPC vaccination, exhibited fevers at 23 DPE (16 days post-first contact, DP1C), and the number of febrile pigs increased from 23–36 DPE. The fever was correlated with CSFV-associated clinical signs that manifested at 24 DPE (17 DP1C). Although one piglet (8138) presented mild clinical signs (scores below 5), the other two piglets clinically deteriorated from 24–36 DPE, with their scores ranging between 12 and 16. Piglet 8136 of Group 3 died at 29 DPE. The Group 4 piglets that were in contact (cohabited) with those of Group 2 were also healthy and did not have fever or CSFV-associated clinical signs during the experimental period. The Group 5 piglets that were in contact with those of Group 3 exhibited fevers at 27 DPE (10 days post-second contact, DP2C) and CSFV-associated clinical signs at 30 DPE (13 DP2C). The number of febrile piglets and the clinical scores in Group 5 increased over time; the score at 39 DPE was 21 ± 1.4, ranging between 19 and 22.

According to the clinical parameters, the presence of MDAs only (Group 3) could not protect the piglets from contact transmission induced by the primary invader (Group 1). A single dose of the LPC vaccine in addition to MDAs (Group 2; i.e., the widely applied practice) offered protection from contact transmission induced by the primary invader (Group 1; Table 1). The widely applied vaccination practice also benefited the secondarily invaded piglets (i.e., Group 4, which only had MDAs but were free of fever and clinical signs). Clinically, the situation in Group 5 (the piglets with contact transmission induced by the secondary invaders) recapitulated the situation in Group 3 (contact transmission induced by the primary invader).

### 3.2. CSFV Viremia

**Figure 2:**
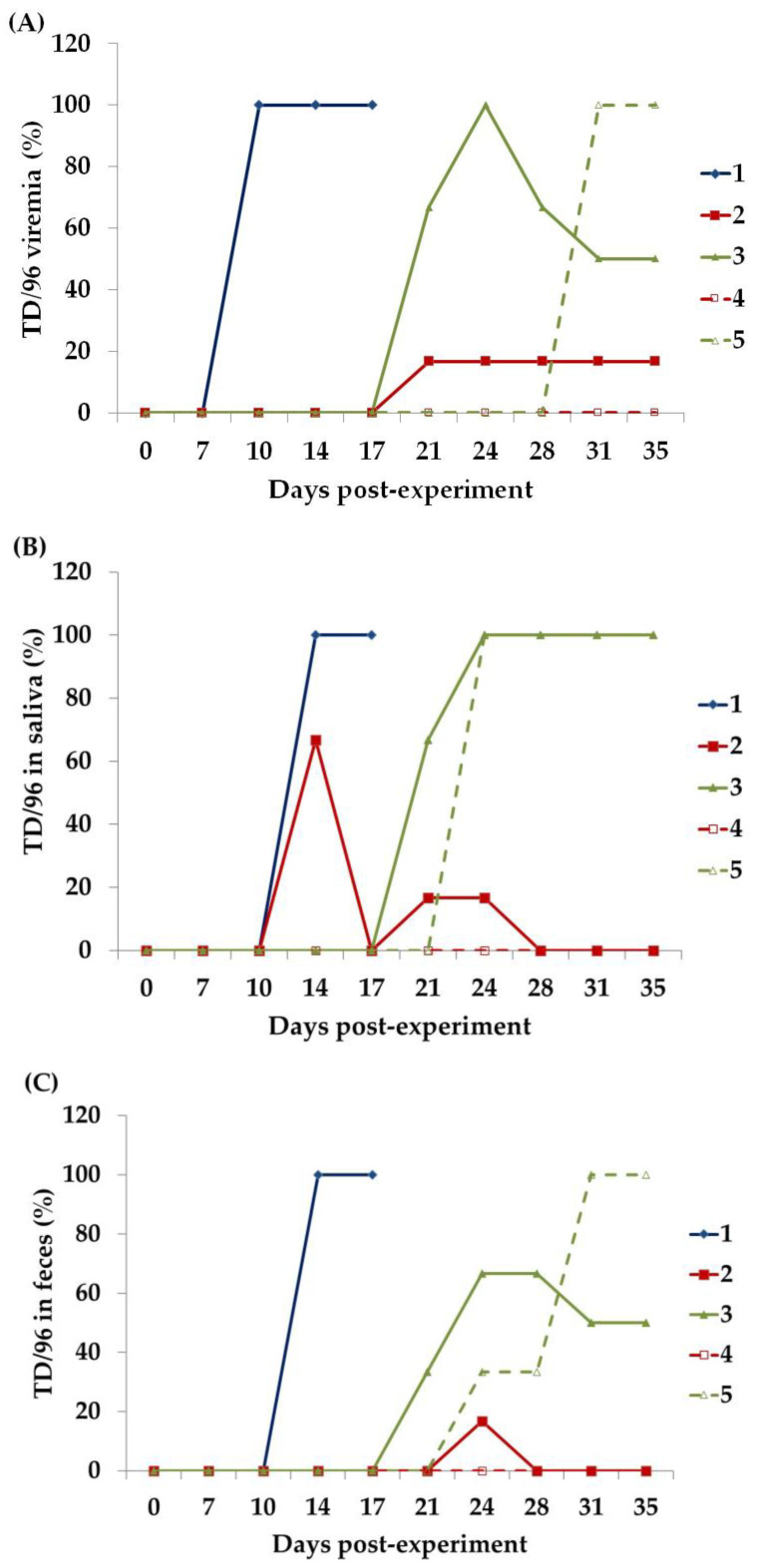
Number (percentage) of piglets positive for TD/96 as detected through QRRT-PCR [20] of the (**A**) blood, (**B**) saliva, and (**C**) feces of the piglets in each group during the experimental period. The Group 1 piglet was inoculated oronasally with TD/96 at 7 DPE and served as the CSFV donor (i.e., primary invader) for Groups 2 and 3. The piglets in Group 2 that underwent LPC vaccination at 0 DPE and those in Group 3 that did not undergo LPC vaccination cohabited with the Group 1 piglet from 7–17 DPE. The piglets in Groups 4 and 5 cohabited with those in Groups 2 and 3 (i.e., secondary invaders), respectively, from 17–36 DPE.

**Figure 3:**
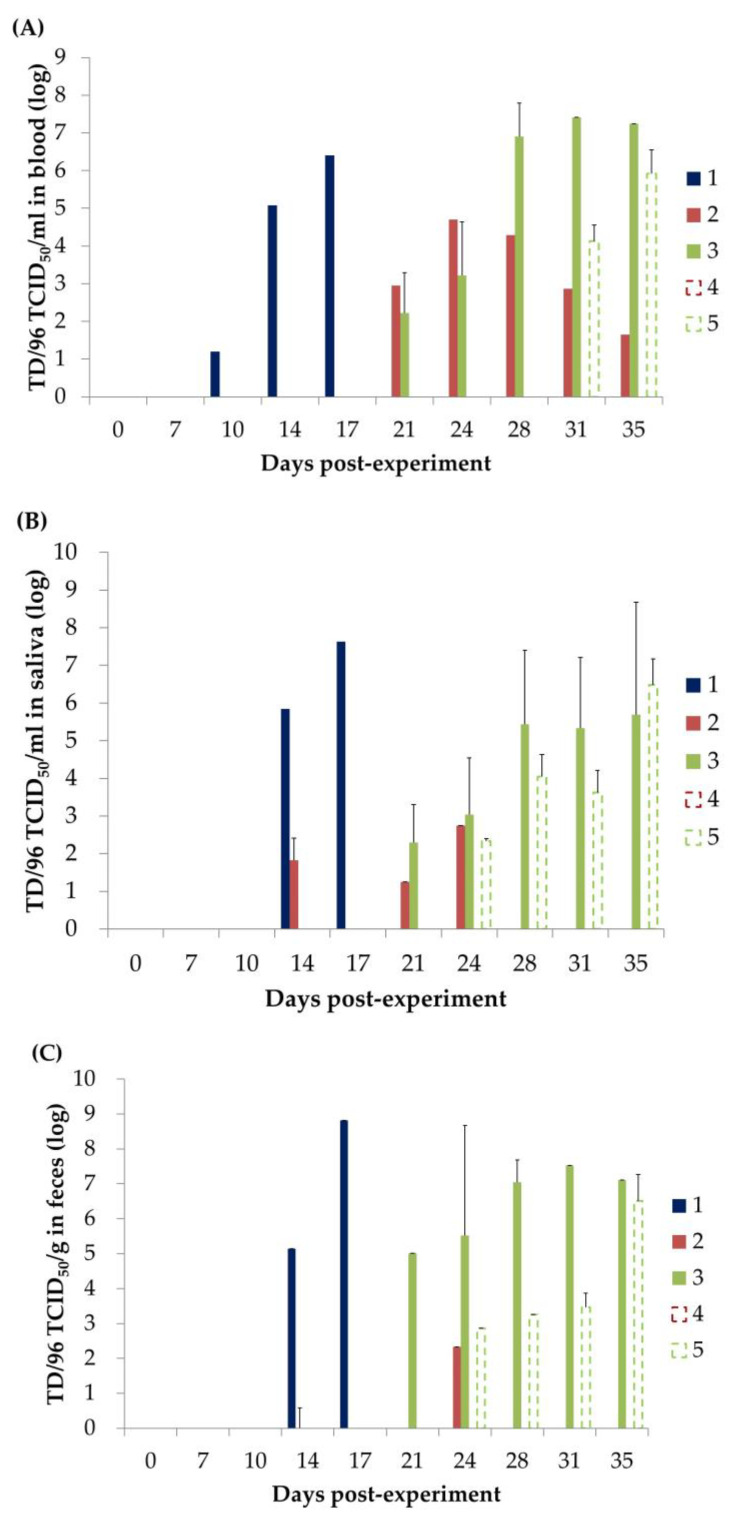
CSFV loads present in the (**A**) blood, (**B**) saliva, and (**C**) feces of the piglets in each group.

The CSFV TD/96 strain was not detected in the blood of any of the piglets before the experiment (Figure 2A and Figure 3A). After TD/96 inoculation, TD/96 viremia in the Group 1 piglet was first detected at 10 DPE (3 days post-inoculation, POI) and continuously detected until 17 DPE. The CSFV loads increased from 10^1.2^ TCID_50_/mL at 10 DPE to 10^6.4^ TCID_50_/mL at 17 DPE. In Group 2, comprising the piglets with LPC vaccination that had cohabited with the primary invader, TD/96 viremia was detected in only one piglet (8134) from 21–35 DPE. The CSFV loads in the blood of Piglet 8134 peaked (10^4.9^ TCID_50_/mL) at 24 DPE and subsequently decreased to 10^1.7^ TCID_50_/mL at 35 DPE. In Group 3, comprising the piglets without LPC vaccination that had cohabited with the primary invader, TD/96 viremia was first detected in 66.7% (2/3) of the piglets at 21 DPE (14 DP1C), and all were positive by 24 DPE. However, Piglet 8138 was negative for TD/96 viremia from 28–35 DPI (Figure 2A). In Group 4, consisting of the piglets that cohabited with Group 2 in Room 2, TD/96 viremia was not detected during the experimental period. All piglets of Group 5, which comprised the piglets that had been in contact with Group 3, were positive for TD/96 viremia from 31–35 DPE. The CSFV loads ranged between 10^5.1^ and 10^6.7^ TCID_50_/mL at 35 DPE (Figure 3A).

In Groups 2 and 3, the presence of MDAs delayed the first detection of viremia until 10 DP1C (i.e., the piglets remained asymptomatic until 17 DPE; Table 1) with the primary invader, as compared with day 3 POI in Group 1 in the acute phase. The additional single dose of the LPC vaccine (Group 2) reduced the percentage of viremic pigs by 83% (Figure 2A). Similarly, the additional single dose of the LPC vaccine reduced the viral loads in the blood from 28–35 DPE (Figure 3A). The LPC vaccination of Group 2 also benefited the Group 4 piglets they subsequently cohabited with (secondarily invaded), despite one of the Group 2 piglets having transient viremia (Figure 2A) and saliva (Figure 2B) and fecal shedding (Figure 2C) of TD/96. This change in the viremic status correlated with that determined on the basis of the clinical parameters (Section 3.2), although the laboratory detection was more sensitive.

### 3.3. CSFV Shedding in Saliva

The CSFV TD/96 strain was not detected in the saliva of any of the piglets before the experiment (Figure 2B and Figure 3B). The saliva of the Group 1 piglet was first positive for TD/96 at 14 DPE (7 DP1C), which continued until 17 DPE. The CSFV loads increased from 10^5.8^ TCID_50_/mL at 14 DPE to 10^7.6^ TCID_50_/mL at 17 DPE. Notably, in the Group 2 piglets that had been vaccinated with LPC and had cohabited with the CSFV donor, TD/96 was first detected in 66.7% (4/6) of the saliva samples at 14 DPE (7 DP1C), which became negative thereafter. Later, another piglet (1/6, Piglet 8134 with TD/96 viremia; Figure 2A), shed TD/96 in its saliva from 21–24 DPE at 10^1.2^ and 10^2.7^ TCID_50_/mL, a lower level than those of the aforementioned piglets. In Group 3 with the piglets with MDAs only and without LPC vaccination that had cohabited with the CSFV donor, TD/96 was initially detected in 66.7% (2/3) of the saliva samples at 21 DPE, and all saliva samples were positive by 24 DPE. In Group 4 comprising the piglets that cohabited with those in Group 2, TD/96 was not detected in the saliva samples during the experiment, whereas in Group 5, comprising the piglets that cohabited with those in Group 3, all saliva samples were positive for TD/96 from 24–35 DPE. The CSFV loads increased over time and ranged from 10^5.9^ to 10^7.3^ TCID_50_/mL by 35 DPE.

A total of four-sixths of the piglets in Group 2 (with single-dose LPC vaccination) had TD/96-positive saliva at 14 DPE before viremia. CSFV was detected earlier in the piglets of Group 2 that had been in contact with the Group 1 piglet during its TD/96 shedding. One piglet (Piglet 8134) in Group 2 later exhibited transient shedding from 21–24 DPE, which is more likely representative of CSFV’s replication in the salivary glands after viremia. According to the comparison between Groups 2 and 3, the LPC vaccination decreased CSFV shedding.

### 3.4. CSFV Shedding in Feces

The CSFV TD/96 strain was not detected in the feces of any of the piglets before the experiment (Figure 2C and Figure 3C). The feces of the Group 1 piglet were TD/96 positive first at 14 DPE (7 DPIC), which continued until 17 DPE. The CSFV loads increased from 10^5.1^ TCID_50_/g at 14 DPE to 10^8.8^ TCID_50_/mL at 17 DPE. In Group 2, comprising the piglets with LPC vaccination that cohabited with the Group 1 CSFV donor, only Piglet 8134 transiently shed at 24 DPE (17 DPIC), whereas all the other piglets in Group 2 were negative. In Group 3, comprising the piglets without LPC vaccination that cohabited with the CSFV donor, two-thirds of the piglets (all except Piglet 8138) were positive between 21 (14 DP1C) and 35 DPE, during which time the CSFV loads increased from 10^5.1^ TCID_50_/g at 21 DPE to 10^7.5^ TCID_50_/mL at 31 DPE. However, the fecal samples of Piglet 8138 with transient TD/96 viremia were not positive for TD/96. In Group 4, consisting of the piglets that cohabited with those in Group 2, all fecal samples were negative for TD/96 during the experimental period. In Group 5, which comprised the piglets that cohabited with Group 3, the number of TD/96-positive fecal samples increased over time from 24–35 DPE, and the loads ranged from 10^5.7^ to 10^7.2^ TCID_50_/g at 35 DPE. In Group 5, the first detection occurred at 24 DPE (7 DP2C), which was similar to that in Group 1 (14 DPE), and the overall profile paralleled that of the viremia (Figure 2A), suggesting that CSFV replicated locally following viremia. As detailed in Section 3.3 and Section 3.4, feces and saliva were the primary vehicles of CSFV contact transmission.

### 3.5. CSFV Loads in Tissues Obtained through Necropsy

Tissues of the Group 1 CSFV donor piglet and of the piglets in Groups 3 and 5 that it cohabited with were TD/96 positive (Table 2). The TD/96 loads in the tissues of the Group 1 piglet ranged between 10^3.76^ and 10^9.37^ TCID_50_/g. In Group 5, comprising the piglets that were in contact with those in Group 3, TD/96 was detected in all piglets and distributed across all tissues, with higher loads in the hematolymphoid organs (tonsils, lymph nodes, and spleen) and those organs with a more abundant blood supply (liver, lungs, heart, and kidneys), as presented in Figure 2A,C. The highest TD/96 loads were in the lymphoid tissues (over 10^9.0^ TCID_50_/g). By contrast, the nonhematolymphoid tissues from the cohabited piglets of Groups 2 and 4, except for Piglet 8134 of Group 2, which had transient TD/96 viremia, were low-level TD/96 positive only in the blood, tonsils, inguinal lymph nodes, and submaxillary lymph nodes, with these levels ranging between 10^1.65^ and 10^3.78^ TCID_50_/g. In Group 3, comprising the piglets without LPC vaccination that had been in contact with the Group 1 CSFV donor, TD/96 was detected in all tissues of two of the three piglets (except for Piglet 8138). Piglet 8138 of Group 3, which had transient TD/96 viremia, had TD/96 in its tonsils, submaxillary lymph nodes, and bronchial lymph nodes, although the viral loads were lower than average, ranging between 10^3.08^ and 10^4.69^ TCID_50_/mL. 

### 3.6. CSFV in the Experimental Environment

The fence, feces on the floor, feed trough, and drinking fountain of each experimental room were tested for TD/96 through QRRT-PCR (Table 3). TD/96 was detected at 14 and 17 DPE in the feces on the floor of Room 12, wherein the Group 1 CSFV donor cohabited with the piglets in Groups 2 and 3. The fence, feed trough, and drinking fountain all tested negative. The samples from Room 2 in which Groups 2 and 4 cohabited were all negative from 21–35 DPE. TD/96 was first detected in the feces on the floor of Room 4 (Groups 3 and 5) at 24 DPE. Subsequently, TD/96 was detected on the fence, feed trough, and drinking fountain of Room 4 from 28–35 DPE.

These results indicate that feces (which could be potentially present in all types of environmental samples) and saliva (which would most likely be present in the feed trough and drinking fountain samples) were the primary vehicles for contact transmission. The transient viral shedding of the Group 2 piglets (Figure 2 and Figure 3) was not introduced into Room 2, where they cohabited with the piglets in Group 4.

### 3.7. CSFV-Specific IFN-γ-Secreting PBMCs

**Figure 4:**
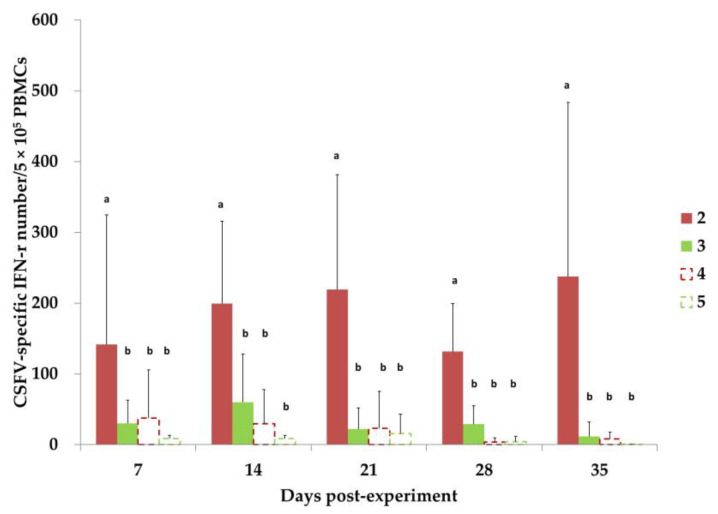
CSFV-specific IFN-γ-secreting cells examined in the PBMCs of piglets in Groups 2 to 5 between 7 and 35 DPE. The piglets in Group 2 with LPC vaccination at 0 DPE and those in Group 3 without LPC vaccination cohabited with the Group 1 CSFV donor piglet from 7 to 17 DPE. The piglets in Groups 4 and 5 cohabited with those in Groups 2 and 3, respectively, from 17 to 35 DPE. Values with different superscript letters, a and b, indicate a statistically significant difference (*p* < 0.05) from each other. No significant differences exist between values containing the same letter.

CSFV-specific IFN-γ-secreting cells were examined in the PBMCs of the piglets in Groups 2 to 5 between 7 and 35 DPE (Figure 4). In the Group 2 piglets, the numbers of IFN-γ-secreting cells, although at a frequency of less than 0.1% of the PBMCs, were significantly higher than those of Groups 3, 4, and 5 between 7 and 35 DPE. The numbers of IFN-γ-secreting cells in Piglet 8134 of Group 2, which had transient TD/96 viremia within a similar timeframe (Figure 2A), correlated with the significantly lower numbers of 56 (21 DPE) and 62 (28 DPE; group average ≥116); however, these numbers reached the group average by 35 DPE. The number of CSFV-specific IFN-γ-secreting cells in Groups 3, 4, and 5 were not significantly different.

### 3.8. Anti-CSFV NAs

**Figure 5:**
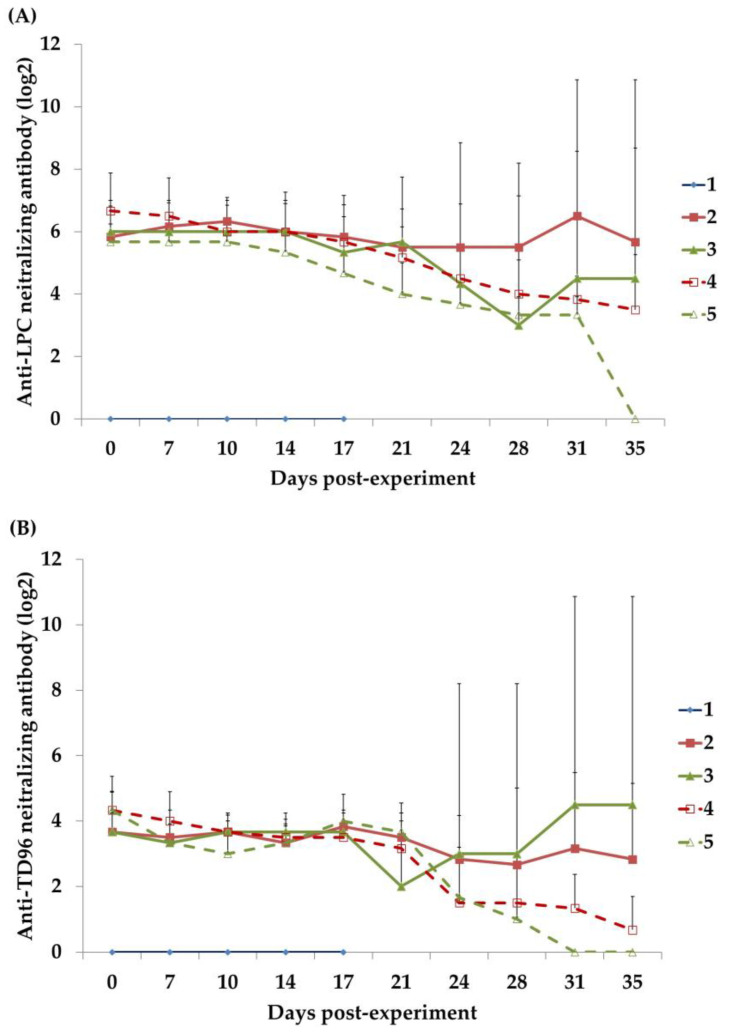
The anti-LPC (**A**) or TD/96 (**B**) NAs in the sera of the piglets in each group during the experimental period. The Group 1 piglet was inoculated oronasally with TD/96 at 7 DPE and served as the CSFV donor (i.e., primary invader), subsequently cohabiting with the piglets of Groups 2 and 3 from 7 to 17 DPE. The Group 2 piglets were LPC vaccinated at 0 DPE, and the Group 3 piglets were not vaccinated with LPC. The piglets in Groups 4 and 5 cohabited with those in Groups 2 and 3, respectively, from 17 to 35 DPE.

The anti-CSFV NAs in the piglet sera were generated in response to either the LPC strain (Figure 5A) or the TD/96 strain (Figure 5B). Anti-CSFV NAs were not detected in the Group 1 CSFV donor between 7 and 17 DPE. The sera of the piglets in Groups 2 to 5 at 0 DPE exhibited titers ranging between 5 and 7 log_2_ (between 32- and 128-fold). After the Group 2 piglets were vaccinated with the LPC vaccine and cohabited with the CSFV donor, the average titer did not decrease between 0 and 28 DPE. Due to the increase in anti-LPC NAs in Piglet 8134 from 31–35 DPE, the average titer of Group 2 significantly increased. In the Group 3 piglets without LPC vaccination that cohabited with the CSFV donor, the average titer gradually decreased between 0 and 28 DPE. As the titer of Piglet 8138 gradually increased between 21 and 35 DPE, the average titer of Group 3 conversely increased between 31 and 35 DPE. The average titers of Groups 4 and 5 decreased over time. In general, the anti-TD/96 NA profile of each group paralleled that of the anti-LPC NA profiles, although the anti-TD/96 NA average was between 1.3 and 3.7 log_2_ (roughly 2–12-fold; Figure 5B), which was lower than the anti-LPC NA average.

The estimated half-life of the MDAs derived from the antibody titers of the Group 4 piglets transferred through the colostrum of LPC-vaccinated sows was 10.7 days (Figure 5A). This estimation is supported by the negative fever and clinical signs (Table 1), the negative viremia, saliva, and fecal viral loads (Figure 2 and Figure 3), and the tissues (Table 2; see Section 3.9) and environmental samples (Table 3) of the Group 4 piglets, which indicated that they had not been infected with the TD/96 virus during the experimental period.

### 3.9. Immunohistochemistry Detecting CSFV Antigen Signals in Necropsy Tissue Samples

**Figure 6:**
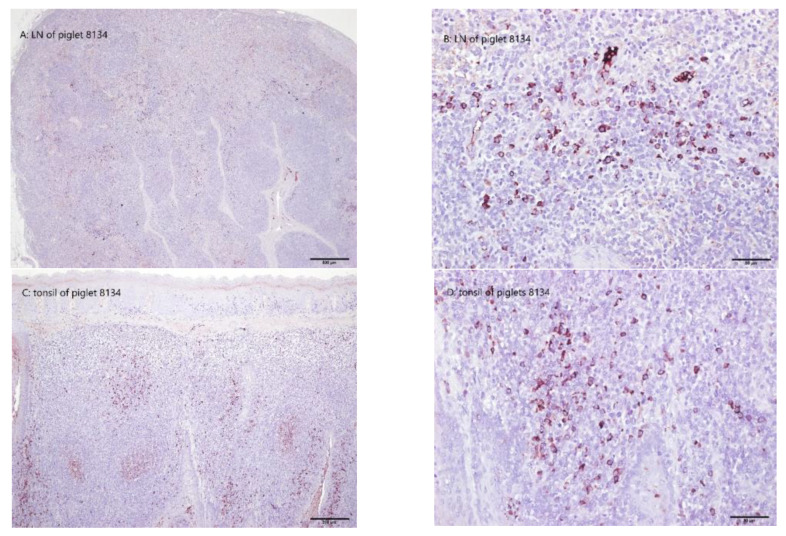

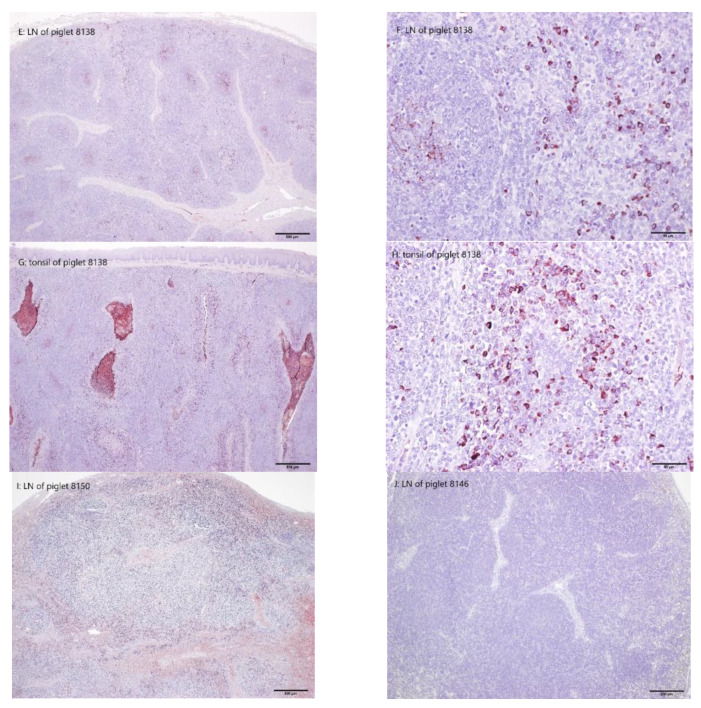
CSFV antigens in the lymphoid tissues were marked by the 1C7A1 monoclonal antibody. A lymph node (**A**,**B**) and tonsil (**C**,**D**) of Piglet 8134 (Group 2), which had transient TD/96 viremia, present the brown TD/96-positive signal. A lymph node (**E**,**F**) and tonsil (**G**,**H**) of Piglet 8138 (Group 3), which had transient TD/96 viremia, present the brown TD/96-positive signal. A lymph node (**I**) of Piglet 8150 (Group 1) presents a diffuse brown TD/96-positive signal in the paracortex. A lymph node (**J**) of Piglet 8146 (Group 4) was negative for TD/96.

The signals of CSFV TD/96 were detected in the lymphoid tissues of the Group 1 CSFV donor (Figure 6I), of one piglet in Group 2 (Piglet 8134, which had CSFV loads in the blood that were at the highest level of 10^4.9^ TCID_50_/mL at 24 DPE and that subsequently decreased to 10^1.7^ TCID_50_/mL at 35 DPE), of three piglets in Group 3, and of three piglets in Group 5. The Group 4 piglets were not positive for TD/96 (Figure 6J). The strong CSFV TD/96 signals were widely distributed in the tonsils, spleens, and lymph nodes of the piglets in Groups 1, 3 (except for Piglet 8138), and 5. For Piglet 8134 (Group 2) with transient TD/96 viremia, scattered TD/96-positive signals were located mostly in the parenchymal regions of the tonsils, medulla of the inguinal lymph nodes, and submaxillary lymph nodes (Figure 6A–D), which is consistent with the results of the viral load quantification of these tissues (Table 2). The morphology and distribution of the TD/96-positive cells were strongly macrophagic. In Piglet 8138 (Group 3) with transient TD/96 viremia, the number and distribution of the TD/96-positive signals (Figure 6E–H) were similar to those in Piglet 8134.

In Piglet 8134 (Group 2) and Piglet 8138 (Group 3), both with transient low-level viremia (Figure 2A and Figure 3A; Table 2), CSFV antigens were still detectable in the tissue macrophages of the tonsils (Figure 6A–H), which again demonstrated that the tonsils are an ideal tissue for CSFV diagnosis.

## 4. Discussion

Vaccination is crucial for CSF prevention and control in CSF-endemic areas. A widely applied practice is to provide neonates with MDAs through the colostrum of vaccinated sows and then to administer a single dose of MLV (such as LPC) when the piglets are aged 3 to 6 weeks; this procedure was employed for Group 2 in the present study (Figure 1). Many factors, including vaccine quality, vaccination program procedures (e.g., schedules, types, and routes), and pigs’ MDA levels and health statuses (including individual variation), can influence the efficacy of CSFV vaccination, reducing protection against infection [14,15,16]. In the present study, although the piglets in Groups 2 and 3 had high MDA levels at 1:32–128-fold at 7 DPE (Figure 5A; i.e., 0 DP1C with the Group 1 CSFV donor; Figure 1), the MDAs delayed the progress of the infection, as indicated by the late appearance of the viremia at 10 DP1C (Groups 2 and 3; Figure 2A). This caused the piglets to be asymptomatic (Table 1), although some of the piglets still shed the virus in their saliva and feces (Figure 2B,C). These occasional viral shedders in Group 2 represented individual variations in the group health statuses or immune responses analyzed in this study. MDAs alone can only offer partial protection before viral replication occurs in the blood and tissue. The anti-CSFV TD/96 NA level was not positively converted following LPC vaccination (Figure 5B), even after the piglets cohabited with the Group 1 primary invader. This likely occurred because the MDA level did indeed interfere with the MLV’s efficacy and because of the immunosuppressive nature of the invading CSFV TD/96.

The titers of the anti-CSFV NAs and CMI represented by the number of CSFV-specific IFN-γ-secreting cells are closely related to the protection of pigs against CSFV infection [17,22,23,24]. Piglet 8134 (Group 2), with low anti-CSFV NA levels and low numbers of CSFV-specific IFN-γ-secreting cells after MLV inoculation (Figure 4 and Figure 5), exhibited transient CSFV viremia and shedding after contact with the CSFV donor (Figure 2 and Figure 3). By contrast, the other piglets in Group 2, with low anti-CSFV NAs but relatively high CSFV-specific IFN-γ-secreting cells following MLV inoculation, were not infected with CSFV, even after cohabiting with the CSFV donor for 10 days. In addition, although Piglet 8138 (Group 3) had a high anti-LPC NA titer (over 128-fold) without MLV vaccination and with minimal CSFV-specific IFN-γ-secreting cells, it also exhibited transient CSFV viremia after cohabiting with the TD/96 donor but was CSFV-free by the end of the experimental period. The other piglets in Group 3, with low anti-LPC NA levels (lower than 64-fold) and without MLV vaccination, presented severe CSFV-associated clinical signs, lesions, and high CSFV tissue loads, which resulted in death. A comparison of the results of Groups 2 and 3, which had similar anti-CSFV titers and profiles (Figure 5), indicates that CMI is the key factor in viral clearance and recovery. The Group 2 piglets had more CMI (Figure 4), which is consistent with the free clinical scores (Table 1) and all the tested parameters (Figure 2, Figure 3, and Figure 6).

Notably, in Group 2 and the other groups, the CMI levels did not markedly increase (and only at a frequency lower than 0.1% of the PBMCs) throughout the experimental period (Figure 4), despite the piglets’ constant exposure to the viral shedding of the Group 1 CSFV donor and the Group 3 piglets from 7–17 DPE (Figure 2 and Figure 3). The low frequencies of IFN-γ-secreting cells likely reflect the following: (1) the immunosuppressive nature of CSFV, (2) MDA interference, and (3) the technical limitation of the assay that we performed. Therefore, determining a suitable window in which the MDA level is sufficiently low to avoid interference and yet sufficiently high to provide initial protection and enhance anti-CSFV CMI is essential for improving the immunity of herds.

For the Group 2 piglets treated with the widely applied vaccination procedure, the occasional shedding of CSFV in saliva and feces occurred for a short period (Figure 2) at low CSFV loads (Figure 3); thus, the piglets in Group 4 (with MDAs only) blocked CSFV transmission to Group 4, as indicated by the CSFV-negative results of the environmental samples collected from Room 2 (Table 3), the lack of fever and clinical signs (Table 1), and the absence of saliva and fecal shedding and viremia (Figure 2 and Figure 3) during cohabitation. However, the asymptomatic CSFV carriers in Group 2 could have been an undetectable liability in the field.

Immune response can be quickly and steadily induced in SPF piglets vaccinated with the MLV. The cellular and humoral immune responses of MLV-vaccinated piglets are revealed at 5 and 12 days post-vaccination, respectively, at the earliest [14,15,16,22]. When the MLV is used in commercial herds, the MDAs reduce the MLV’s efficacy. In the present study, the CMI of the Group 2 piglets with substantial MDA levels increased rapidly at 0 DP1C (7 DPE; Figure 4; i.e., much quicker than in SPF pigs without MDAs), although neither the antibody nor CMI profiles changed dramatically (Figure 4 and Figure 5). Such a quick increase at 7 DPE (Figure 4) after LPC vaccination suggests that the colostrum may provide other immunological components, in addition to MDAs, that induce an anamnestic-like response.

Anti-CSFV NAs are commonly used to evaluate vaccine efficacy and to survey for CSFV infection in herds. According to the CSFV strain, the titer of the anti-CSFV NAs against homologous strains (e.g., LPC of the genotype 1.1) is higher than that against heterogeneous strains (e.g., TD/96 of the genotype 2.1) and, in this study, was log_2_ 1.3 to 3.7 (Figure 5A) higher than that against the TD/96 strain (Figure 5B) [14]. A similar NA titer difference was also observed in different strains of the porcine reproductive and respiratory syndrome virus (PRRSV) and SARS-CoV-2 [25,26]. As MDAs are a vital interference factor for MLV efficacy, the optimal vaccination schedule for MLV inoculation in piglets is when the MDAs of the piglets are lower than 1:32-fold of the anti-LPC NA titer. Therefore, the optimal MLV vaccination time in the present study was at 28 DPE, which is consistent with our estimation that the half-life of the MDAs was 10.7 days (Group 2; Figure 5A) and similar to the analysis results of the anti-LPC NA titers of Groups 4 and 5. Paradoxically, the 32-fold level of the anti-CSFV NA titer (roughly equivalent to 1:128-fold of anti-LPC NAs) is considered the protective index for CSFV infection [17]. Most of the piglets in Groups 4 and 5 had less than 1:32-fold of anti-TD/96 NA titers from 7 DPE onward. Thus, the window period when MDA levels are suitably low to not interfere with MLV vaccination but sufficiently high to be protective is narrow, whereas the window for the risk of CSFV infection is wide. The suitable period for MLV vaccination can be extended only if the MLV is produced from homologous strains, which are usually unavailable in most CSFV-endemic areas.

Herd immunity negatively correlates with pathogen spread in the prevention and control of diseases. Increased herd immunity can reduce both the susceptibility to infection and the magnitude of the pathogen shedding in the herd [27,28]. Vaccination is commonly used as a direct and quick tool to increase herd immunity. Improving herd immunity coverage through vaccine immunization to reduce pathogen spread has been demonstrated to be effective for the control of PRRSV, porcine circovirus type 2, CSFV, pseudorabies virus, and SARS-CoV-2 [29,30,31,32,33,34,35,36]. In CSFV studies, the administration of both MLV and E2 subunit vaccines also reduced CSFV transmission in herds [14,15,16,22,32]. In the present study, although a few MLV-vaccinated piglets exhibited transient CSFV viremia and shedding (Groups 2 and 3; Figure 2 and Figure 3), MLV inoculation not only offered complete protection to the vaccinated pigs but also ensured that the TD/96 strain was not transmitted from the primary invader (Group 1) to the secondarily invaded group (Group 4). By contrast, TD/96 spread from the Group 1 CSFV donor to Group 3 (without MLV vaccination) and over to Group 5, demonstrating that MLV vaccination against CSFV can block CSFV transmission, thus increasing the probability of CSFV eradication by reducing the value of the basic reproduction number (R0) of the pathogen [28]. More extensive vaccine coverage is required to eradicate diseases with high-R0 pathogens. The R0 of CSFV is associated with the strain virulence and CSFV inoculation dose. The R0 pathogen levels of moderate-inoculation and high-inoculation doses of a moderately virulent strain and the low-inoculation dose of a highly virulent strain are substantially higher than that of a high-inoculation dose of a low-virulence strain [13]. These results indicate that more herd immunity coverage is required in CSF-endemic areas with moderate or highly virulent CSFV strains.

Monocyte–macrophage lineage cells are CSFV tropism cells that play a role in CSFV transmission [37,38,39]. In the present study, the piglets that recovered were healthy and asymptomatic; however, CSFV can persistently infect and hide in lymphoid tissues (Figure 6), thus avoiding immune clearance; this also supports the use of lymphoid tissues as ideal specimens for diagnosis. Persistent viral infection has also been observed with CSFV, human immunodeficiency virus type 1, and respiratory syncytial virus [40,41,42]. CSFV has the capacity to alter the expression of cytokines, cytokine receptors, chemokines, interferons, and toll-like receptors in macrophages, reflecting its immunosuppressive nature and the near inactivity of the antibody (Figure 5) and CMI (Figure 4) profiles of the experimental pigs, despite their continual exposure to CSFV shedding in the saliva and feces of the primary (Group 1) and secondary (Group 3) invaders. The immunoregulation of the pigs after CSFV infection involved increases in proinflammatory (IL-1, IL-6, IL-8, and MCF) and antiviral factors (IFN-α and β) [43]. However, the evolved mechanisms and persistent infection of CSFV remain unclear.

## 5. Conclusions

MDAs followed by MLV vaccination can induce sufficient immunity, particularly CMI, enabling viral clearance and recovery. Although a few MLV-vaccinated piglets had transient low-level viremia and viral shedding in their saliva and feces, CSFV transmission to the third party (Group 4) could be blocked through MLV vaccination, thus reducing the R0 of CSFV and increasing the possibility of CSF eradication.

## Figures and Tables

**Table 1:** Percentage of febrile piglets in each group during the experimental period.

Days Post-experiment (DPE)	0	7	8	9	10	11	12	13	14	15	16	17	18	19	20	21	22	23	24	25	26	27	28	29	30	31	32	33	34	35	36	37	38	39
Days post-first contact (DP1C)		0	1	2	3	4	5	6	7	8	9	10	11	12	13	14	15	16	17	18	19	20	21	22	23	24	25	26	27	28	29	30	31	32
Days post-second contact (DP2C)												0	1	2	3	4	5	6	7	8	9	10	11	12	13	14	15	16	17	18	19	20	21	22
Group 1 (*n* = 1)	0	0	0	0	0	0	100	100 ^#^	100	100	100	100	100																					
Group 2 (*n* = 6)	0	0	0	0	0	0	0	0	0	0	0	0	0	0	0	0	0	0	0	0	0	0	0	0	0	0	0	0	0	0	0			
Group 3 (*n* = 3)	0	0	0	0	0	0	0	0	0	0	0	0	0	0	0	0	0	33	33 ^#^	33	100	100	100	100	100	100	100	100	100	100	100			
Group 4 (*n* = 6)	0	0	0	0	0	0	0	0	0	0	0	0	0	0	0	0	0	0	0	0	0	0	0	0	0	0	0	0	0	0	0	0	0	0
Group 5 (*n* = 3)	0	0	0	0	0	0	0	0	0	0	0	0	0	0	0	0	0	0	0	0	0	33	33	33	67 ^#^	67	67	100	100	100	100	100	100	100

^#^ Indicates the day and the number (percentage) of piglets presenting CSF-associated clinical signs in each group. The CSF-associated clinical signs were observed in piglets in Groups 1, 3, and 5, and only one piglet (Group 3) died at 29 DPE.

**Table 2:** CSFV loads detected in various tissues of the piglets through CSFV QRRT-PCR.

Group	1	2	3	4	5
Serum	5.08 (1/1) ^#^	- (0/6)	7.52 ± 0.39 (2/3) *	- (0/6)	5.91 ± 0.78 (3/3)
EDTA: blood	6.40 (1/1)	1.65 (1/6)	7.59 ± 0.61 (2/3)	- (0/6)	7.63 ± 0.39 (3/3)
Tonsil	7.08 (1/1)	3.78 (1/6)	7.63 ± 2.55 (3/3)	- (0/6)	9.36 ± 0.29 (3/3)
Inguinal lymph node	7.92 (1/1)	2.41 (1/6)	9.50 ± 0.34 (2/3)	- (0/6)	9.45 ± 0.55 (3/3)
Submaxillary lymph node	8.16 (1/1)	3.33 (1/6)	7.79 ± 2.87 (3/3)	- (0/6)	9.63 ± 0.31 (3/3)
Bronchial lymph node	8.10 (1/1)	- (0/6)	7.20 ± 3.60 (3/3)	- (0/6)	9.71 ± 0.35 (3/3)
Lung	7.08 (1/1)	- (0/6)	8.41 ± 1.25 (2/3)	- (0/6)	9.03 ± 0.11 (3/3)
Spleen	9.37 (1/1)	- (0/6)	7.50 ± 1.15 (2/3)	- (0/6)	8.66 ± 0.34 (3/3)
Liver	8.57 (1/1)	- (0/6)	8.71 ± 0.20 (2/3)	- (0/6)	7.87 ± 0.70 (3/3)
Heart	8.17 (1/1)	- (0/6)	9.06 ± 0.13(2/3)	- (0/6)	7.27 ± 0.50 (3/3)
Kidney	8.45 (1/1)	- (0/6)	9.53 ± 0.28 (2/3)	- (0/6)	8.58 ± 1.07 (3/3)
Mesentery lymph node	8.45 (1/1)	- (0/6)	8.92 ± 0.86 (2/3)	- (0/6)	8.75 ± 0.95 (3/3)
Cerebrum	7.65 (1/1)	- (0/6)	8.38 ± 0.40 (2/3)	- (0/6)	7.09 ± 1.31 (3/3)
Cerebellum	8.07 (1/1)	- (0/6)	7.86 ± 1.54 (2/3)	- (0/6)	8.22 ± 0.74 (3/3)
Testes	8.47 (1/1)	- (0/6)	8.06 ± 1.96 (2/3)	- (0/6)	8.16 ± 0.57 (3/3)
Stomach	8.67 (1/1)	- (0/6)	7.46 ± 0.04 (2/3)	- (0/6)	7.60 ± 0.09 (3/3)
Small intestines	3.76 (1/1)	- (0/6)	6.48 ± 1.31 (2/3)	- (0/6)	7.02 ± 0.39 (3/3)
Large intestines	6.88 (1/1)	- (0/6)	8.62 ± 0.65 (2/3)	- (0/6)	7.48 ± 0.30 (3/3)
Bladder	8.51(1/1)	- (0/6)	8.73 ± 0.05 (2/3)	- (0/6)	7.96 ± 0.70 (3/3)

^#^ TD/96 loads of CSFV-positive pigs (CSFV-positive number/total number). The unit of CSFV loads was TCID_50_/g (log). The CSFV loads were calculated for all TD/96-positive tissues. * Piglet 8136 in Group 3 died at 29 DPE. The other piglets in Group 3 were necropsied at 36 DPE. CSFV was detected in the tonsils, submaxillary lymph nodes, and bronchial lymph nodes of Piglet 8138.

**Table 3:** CSFV loads of environmental samples from the experimental rooms.

Room Number	Room 12		Room 2					Room 4				
DPE	14	17	21	24	28	31	35	21	24	28	31	35
Fences	- *	-	-	-	-	-	-	-	-	1.60	1.21	0.94
Feces on floor	0.75 ^†^	2.45	-	-	-	-	-	-	3.48	3.74	3.50	3.25
Feed trough	-	-	-	-	-	-	-	-	-	1.59	2.40	2.78
Drinking fountain	-	-	-	-	-	-	-	-	-	2.45	1.00	3.34

The piglets of Groups 1, 2, and 3 cohabited in Room 12 from 7–17 DPE. The piglets of Groups 2 and 4 cohabited in Room 2 from 17–35 DPE. The piglets of Groups 3 and 5 cohabited in Room 4 from 17–35 DPE. * ‘-’ indicates negative results obtained through QRRT-PCR. ^†^ The unit of value was TCID_50_/g (log).

## Data Availability

The data presented in this study are available upon request from the corresponding authors.
